# Beyond the hallmarks of cancer: enabling technologies reshaping cancer diagnosis, prevention, and treatment

**DOI:** 10.3389/or.2026.1804447

**Published:** 2026-08-04

**Authors:** Sarfaraz K. Niazi

**Affiliations:** College of Pharmacy and Pharmaceutical Sciences, Washington State University, Spokane, WA, United States

**Keywords:** AI-enabled cancer diagnostics, antibody and radioligand-targeted therapeutics, convergent cancer technologies, immune checkpoint and cellular therapies, mRNA neoantigen vaccines, multi-omic liquid biopsy, photonic and photoimmunologic cancer therapies

## Abstract

Cancer biology has been organized for 2 decades by the hallmarks framework, articulated in 2000 and most recently revised in 2026. The pace of technological change in oncology now requires reframing how cancer is detected, prevented, and treated. This review argues that the hallmarks framework, while historically essential, is no longer sufficient on its own to capture contemporary cancer care, and repositions the discussion around enabling technologies that operate across and beyond individual hallmarks. For diagnosis, multi-omic liquid biopsy, AI-assisted radiomics and digital pathology, spatial transcriptomics, single-cell sequencing, and machine-learning analysis of plasma proteomics now support earlier detection, prediction of cancer risk years before diagnosis, monitoring of clonal evolution, and prediction of treatment response. For prevention, the validated population-level success of Human papillomavirus and HBV vaccination is contrasted with investigational directions including mRNA platforms, CRISPR-enabled antigen optimization, personalized neoantigen prophylaxis, and biomarker-stratified molecular interception, none of which has yet been validated in prospective human cancer-prevention trials. For treatment, the discussion is organized by modality class: immune checkpoint inhibitors and resistance strategies, antibody-drug conjugates, bispecific antibodies and T-cell engagers, radioligand therapies, tumor-infiltrating lymphocyte therapy, CRISPR-edited CAR-T and TCR therapies, photonic and photoimmunologic therapies, RNA therapeutics, and adaptive combination regimens. Comparative tables summarize diagnostic platforms, immunotherapeutic modalities, photonic therapies, CRISPR-edited immunotherapy trials, neoantigen vaccine pipelines, and convergent strategies. The trajectory of oncology will be defined less by incremental refinement of hallmarks and more by convergent technologies that integrate diagnostics, prevention, and treatment into continuous, data-driven systems.

## 1 Introduction: from biological taxonomy to technological convergence

Cancer remains the second leading cause of death globally, accounting for approximately 10 million deaths annually [1]. The conceptual framework of cancer hallmarks, first proposed by Hanahan and Weinberg [2] and iteratively refined [3–5], has served as a foundational, tumor-agnostic organizing principle for understanding the multifaceted biology of neoplastic disease. The most recent iteration organizes the field into four interconnected dimensions, recognizing nine hallmark functional capabilities, five enabling phenotypic characteristics, and seven classes of hallmark-facilitating cells within the tumor microenvironment, alongside systemic interactions including aging, obesity, and microbiome dysbiosis that modulate cancer development and progression.

While the hallmarks framework has demonstrated considerable resilience, the most groundbreaking developments in oncological treatment currently originate from enabling technologies. These encompass molecular diagnostics, artificial intelligence, gene editing, immunoprevention, immune checkpoint modulation, antibody engineering, radioligand targeting, computational drug design, and photonic and photoimmunologic therapies. Such technologies are independent of particular hallmark categories, thereby transforming the classification, detection, risk stratification, and management of cancer at every stage of its progression.

**FIGURE 1:**
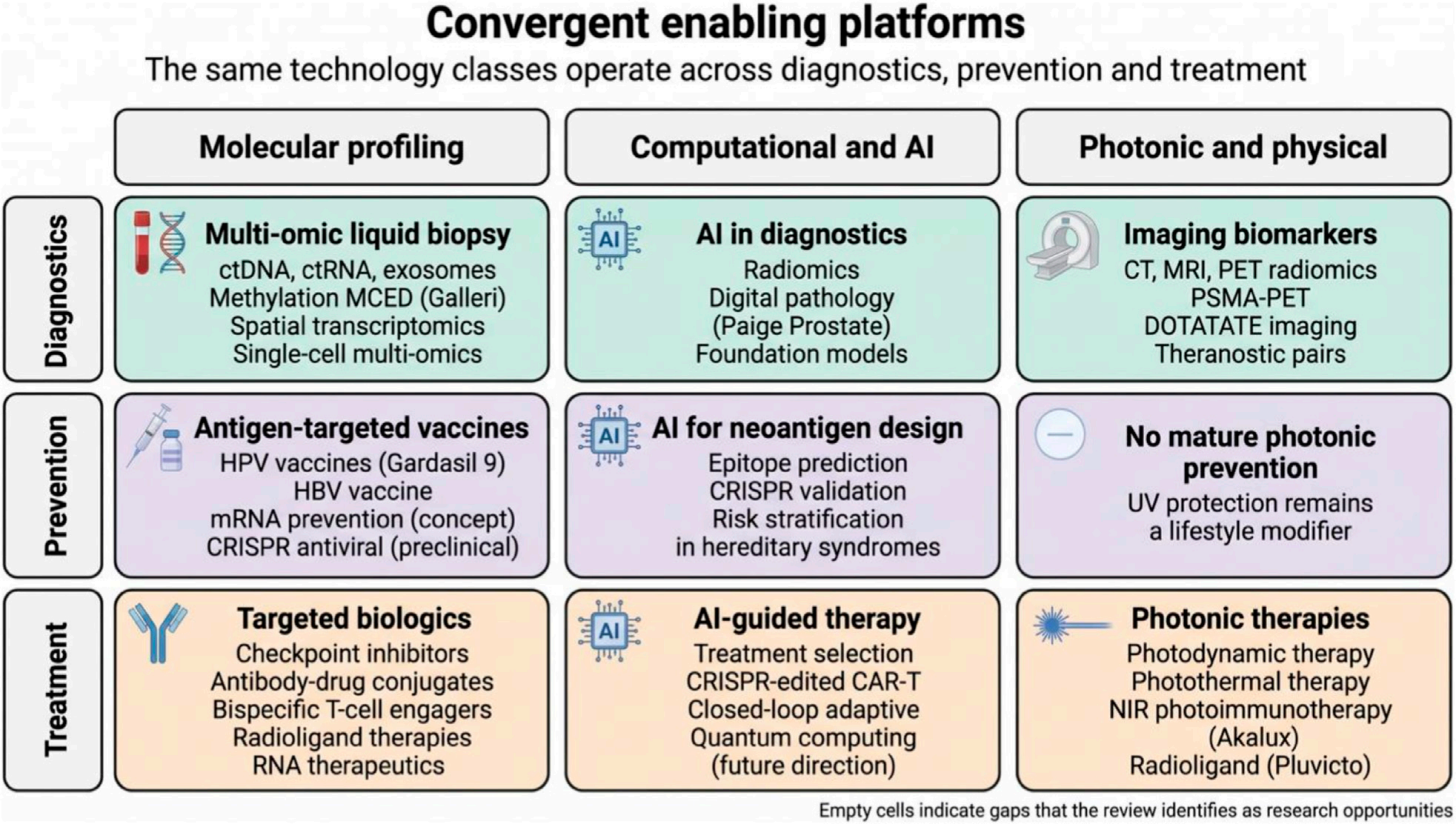
Convergent enabling platforms in cancer care. The diagram systematically categorizes the technologies discussed in this review within a,3-by-3 grid, where rows represent the three intervention domains (diagnostics, prevention, treatment) and columns denote the three classes of enabling platforms (molecular profiling; computational and AI tools, photonic and physical platforms). Each cell enumerates the specific technologies, products, and concepts addressed at that intersection. The diagnostics row encompasses multi-omic liquid biopsy methods (ctDNA, ctRNA, exosomes, methylation MCED), AI-based imaging and pathology tools (radiomics, digital pathology, foundation models), and imaging biomarkers (CT, MRI, PET radiomics; PSMA-PET; DOTATATE imaging; theranostic pairs). The prevention row includes antigen-targeted vaccines (HPV, HBV, and mRNA-based vaccines), AI-driven neoantigen design, and a deliberately sparse photonic prevention cell. The treatment row features targeted biologics (checkpoint inhibitors, antibody-drug conjugates, bispecific T-cell engagers, radioligand therapies, RNA therapeutics), AI-guided therapies, and CRISPR-edited cellular therapies, as well as photonic therapies (PDT, PTT, NIR-PIT, Akalux, Pluvicto). Cells with minimal content, notably photonic prevention, identify gaps in research opportunities as highlighted by the review. The color coding indicates diagnostics in teal, prevention in purple, and treatment in coral, with the same scheme maintained in Figures 2, 3. Created using BioRender. Niazi, S. (2026) https://BioRender.com/q0lvwth.

This review is organized around three technology-driven paradigms. First, next-generation diagnostics that redefine how cancer is detected and classified. Second, immunoprevention strategies, anchored by the established success of HPV and HBV vaccination, with mRNA- and gene-editing-based approaches discussed as future directions. Third, modality-class treatment approaches, including established immunotherapies (immune checkpoint inhibitors, antibody-drug conjugates, bispecific antibodies, radioligand therapies), adoptive cellular therapies (CAR-T, TCR, tumor-infiltrating lymphocytes), CRISPR-edited immune engineering, photonic and photoimmunologic therapies, RNA therapeutics, and adaptive combination regimens that cut across biological mechanisms. Technological convergence, rather than biological taxonomy, now defines the frontier of cancer care. Figure 1 summarizes the convergence framework as a three-by-three grid in which the same enabling platforms (molecular profiling, computational and AI tools, and photonic and physical platforms) operate across diagnostics, prevention, and treatment.

### 1.1 Scope and evidentiary hierarchy

**FIGURE 2:**
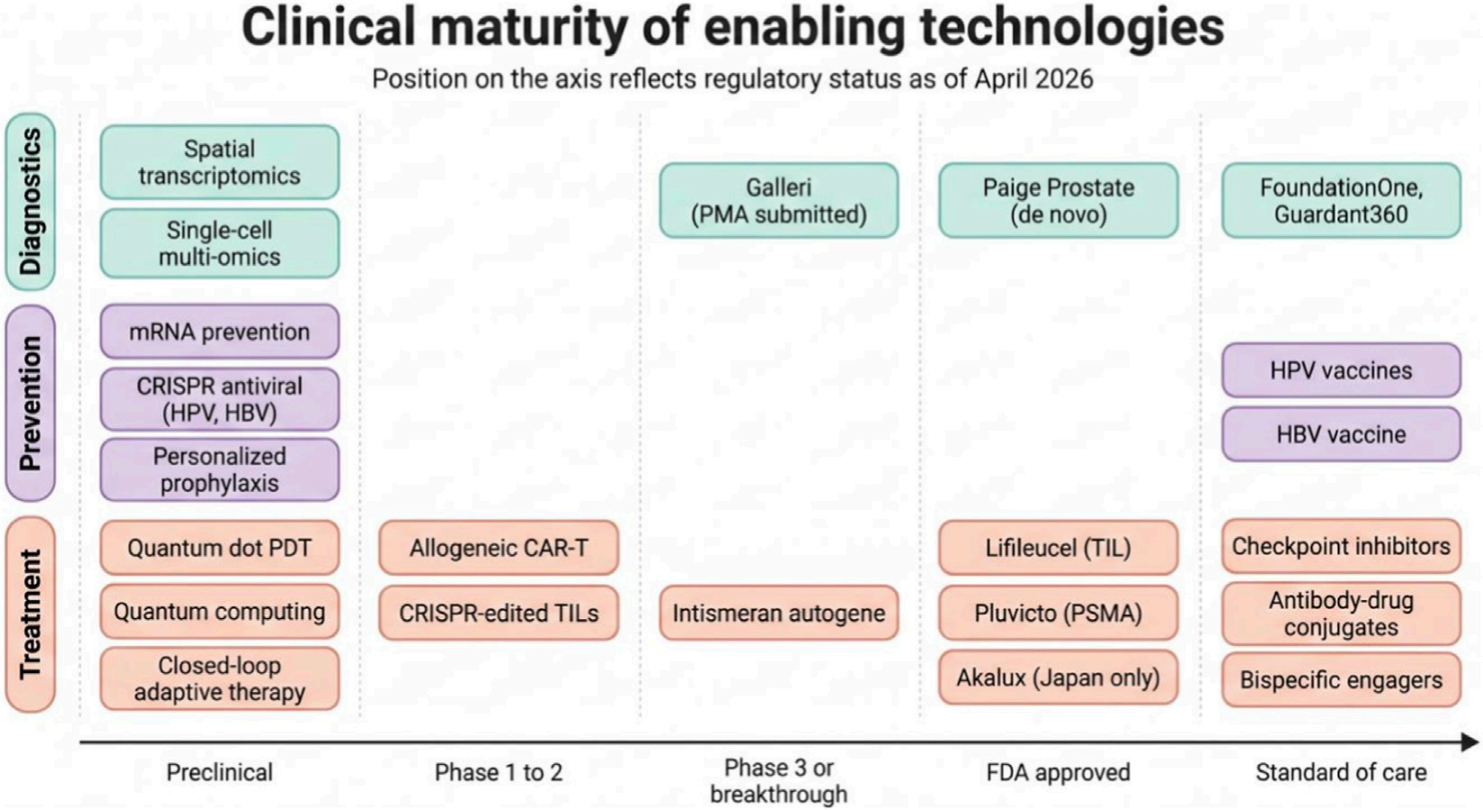
Clinical Maturity of Enabling Cancer Technologies. The horizontal axis delineates the progression of regulatory and clinical maturity, from preclinical concept (left) through early-phase clinical investigation, Phase 3 or breakthrough designation, FDA approval, and reaching routine standard of care (right). Each discussed modality in this review is represented as a labeled tile positioned at its current stage on this axis as of April 2026. Color coding indicates the intervention domain (teal for diagnostics, purple for prevention, and coral for treatment), as shown in Figure 1. Tiles exhibiting reduced fill saturation denote technologies at preclinical or early-phase stages; those with full fill saturation indicate technologies in Phase 3, FDA approval, or in routine clinical use. This figure provides a visual summary, enabling the reader to quickly ascertain, without consulting the regulatory-status footnotes of Table 4 through 7, which technologies are presently in use (HPV and HBV vaccines, FoundationOne and Guardant360 companion diagnostics, checkpoint inhibitors, antibody-drug conjugates, bispecific engagers, lifileucel, Pluvicto), which are at the regulatory frontier (Galleri PMA submitted, Akalux conditional approval in Japan, intismeran autogene Phase 2 b with Phase 3 enrolling), and which remain conceptual or preclinical (mRNA cancer prevention, CRISPR antiviral cancer prevention, quantum-dot photosensitizers, quantum computing for drug design, fully closed-loop adaptive therapy). The figure complements the data-source grading defined in this section and applied throughout the manuscript in the table footnotes. Created using BioRender. Niazi, S. (2026) https://BioRender.com/q0lvwth.

The evidence referenced in this review originates from three categories of sources, listed in descending order of significance. Peer-reviewed primary publications and comprehensive Phase 3 reports serve as the primary foundation for any assertions regarding clinical efficacy or regulatory status. Conference abstracts, oral presentations, and preprints are utilized to supplement peer-reviewed evidence and to indicate the direction of ongoing research. Press-release data and company-reported information are employed solely to illustrate emerging trends and timelines and are not used to substantiate definitive efficacy conclusions. Each table in this review includes a standardized footnote that delineates the data-source categories (peer-reviewed, conference-reported, company-reported, trial-registry) and their respective limitations in terms of evidentiary strength. References that have not been independently verified during this review are restricted to providing supporting context and do not substantiate primary efficacy or regulatory claims. When citing regulatory status, terminology aligns with the definitions provided in Section 3.4. Figure 2 positions each modality discussed in this review on a singular horizontal axis, ranging from preclinical concepts to standard of care, thereby enabling the reader to readily assess the regulatory and clinical maturity of each technology covered.

### 1.2 Article type, methodology, and limitations

This article is a narrative review and is submitted as such. It is not a systematic review or a meta-analysis, and it does not follow the PRISMA reporting framework. The literature was identified through targeted searches of PubMed, Google Scholar, ClinicalTrials.gov, the U.S. FDA approvals database, communications from the European Medicines Agency and the Pharmaceuticals and Medical Devices Agency, the proceedings of the American Society of Clinical Oncology, the European Society for Medical Oncology, the American Association for Cancer Research, and the American Society of Hematology, and selected company press releases for regulatory milestones occurring between January 2020 and April 2026. Earlier foundational publications were retained, as they remained the primary references for concepts that have not been superseded. Search terms combined hallmark-related keywords (for example, “cancer hallmarks” and “tumor microenvironment”) with technology-specific keywords (for example, “liquid biopsy,” “multi-cancer early detection,” “CRISPR CAR-T,” “antibody-drug conjugate,” “bispecific T-cell engager,” “radioligand therapy,” “mRNA cancer vaccine,” “photodynamic therapy,” and “photoimmunotherapy”) and clinical-development descriptors (for example, “Phase 3,” “FDA approval,” “accelerated approval,” “breakthrough device,” and “PMA submission”). Peer-reviewed primary publications and Phase 3 reports were prioritized for any claim of clinical efficacy or regulatory status, with conference abstracts, preprints, and company-reported data identified explicitly when used, in accordance with the evidentiary hierarchy described in Section 1.1.

Several limitations follow from this design and from the state of the field, and the reader should keep them in mind when interpreting the discussion that follows. First, source selection reflects the author’s reading of the field rather than a prespecified search protocol, and selection bias is therefore possible. Second, the field surveyed evolves rapidly, and several of the most important data points cited (the 5-year KEYNOTE-942 update, the GRAIL Galleri PMA submission, and the published [5] hallmarks update) are dated to early 2026 and may be superseded between manuscript acceptance and publication. Third, several efficacy claims drawn from CRISPR-edited cellular therapies, photoimmunotherapy combinations, and individualized neoantigen vaccines rely on press releases, conference abstracts, or single-arm Phase 1 or Phase 1/2 cohorts. These are explicitly identified as such in Tables 1 and 2 and are presented in the text as preliminary signals of feasibility rather than as evidence of established efficacy. The reader should treat such claims as provisional until confirmed in peer-reviewed Phase 3 trials. Fourth, the regulatory landscape for AI/ML-based clinical decision support, multi-cancer early detection, closed-loop adaptive therapy, and gene-editing-based therapeutics is in active flux as of the date of this review, and specific regulatory designations and approvals cited here may change. Fifth, several of the speculative integration scenarios discussed in Table 3 are conceptual or preclinical and have not been tested in humans. The discussion in Section 6 should be read against the backdrop of these limitations (Figure 1).

**TABLE 1:** Selected CRISPR-edited CAR-T cell clinical trials in cancer (2019–2025).

Product	Target	CRISPR edits	Cancer type	Phase	Key results	Data source
CTX110	CD19	TRAC, B2M knockout	B-cell lymphoma	1/2	Favorable safety in allogeneic setting	Company-reported (press release)
CTX130	CD70	TRAC, B2M knockout	Renal cell carcinoma	1	Complete remission longer than 3 years in single patient	Company-reported (press release)
CTX131	CD70	Multiple edits	Kidney, cervical, pancreatic, lung	1/2	Enrolling 2025	Company-reported
CB-010	CD19	PD-1 knockout plus TRAC knockout	Relapsed B-cell NHL	1	94 percent ORR, 69 percent CR (n = 16 evaluable)	Company-reported (press release)
CRISPR-PD1 TILs	Patient-specific	PD-1 knockout	Refractory solid tumors	1	Feasible, well-tolerated in early-phase studies	Peer-reviewed phase 1 reports
neoTCR-P1	Neoantigen-specific	TRAC/TRBC knockout plus TCR knock-in	Multiple solid tumors	1	16 patients treated; feasibility demonstrated	Peer-reviewed phase 1
RHOG/FAS knockout CAR-T	Various	RHOG plus FAS double knockout	Preclinical (multiple)	Preclinical	Enhanced antitumor activity *in vivo*	Peer-reviewed [52]

Regulatory status reflects information available as of the date of this review (April 2026). “PMA, submitted” indicates that a Premarket Approval application has been filed but is not equivalent to approval; FDA, review remained pending as of the date of this review. Plasma proteomic risk-prediction signatures differ from multi-cancer early detection assays in that they estimate future cancer risk years before diagnosis from host and microenvironmental protein patterns rather than detecting tumor-derived material; no such signature is yet approved for clinical use. Abbreviations: cfDNA, cell-free DNA; CNA, copy number alteration; ctDNA, circulating tumor DNA; H&E, hematoxylin and eosin; MCED, multi-cancer early detection; MRD, minimal residual disease; MSI, microsatellite instability; PMA, premarket approval; TME, tumor microenvironment.

**TABLE 2:** Leading personalized neoantigen cancer vaccine platforms (2023–2026).

Platform	Developer	Cancer type	Phase	Key efficacy data	Data source
Intismeran autogene (mRNA-4157, V940)	Moderna and merck	Melanoma (resected stage III/IV)	3 (enrolling)	Phase 2 b 5-year update (n = 157): 49 percent reduction in recurrence or death (HR 0.510, 95 percent CI 0.294 to 0.887, one-sided nominal p = 0.0075; company disclosure, peer-reviewed analysis pending) versus pembrolizumab alone. Earlier 3-year analysis reported in conference abstract form 44 percent recurrence reduction and 65 percent distant metastasis reduction [67]	Press release January 2026 for 5-year data (peer-reviewed publication pending); peer-reviewed phase 2 b primary analysis ([68], lancet); 3-year update conference abstract [67]
Autogene cevumeran (BNT122)	BioNTech and genentech	Pancreatic ductal adenocarcinoma (resected)	1/2	Phase 1 (n = 16): 8 of 16 patients had vaccine-induced T-cell responses; responders had longer recurrence-free survival at 3.2-year median follow-up (median not reached in responders versus 13.4 months in non-responders, p = 0.007)	Peer-reviewed ([69], nature; [70], nature)
BNT111	BioNTech	Melanoma	2	BNT111 plus cemiplimab met its primary endpoint in the randomized phase 2 BNT111-01 trial (NCT04526899), with an ORR of 18.1 percent in anti-PD-(L)1 relapsed or refractory advanced melanoma; topline only, full data pending peer-reviewed publication	Company-reported (press release [71]); conference data pending peer-reviewed publication
TG4050	Transgene	HNSCC, ovarian	1	No recurrences reported in vaccinated arm at 18-month follow-up; 30 personalized neoantigens encoded	Conference data (peer-reviewed publication pending)
CVGBM	CureVac	Glioblastoma	1	*De novo* T-cell responses reported in approximately 77 percent of treated patients	Conference data (peer-reviewed publication pending)
Personalized colorectal mRNA platform	Academic and NHS partners	Colorectal cancer	1 (recruiting)	Recruiting as of mid-2024; no efficacy data available	Trial registry

All entries reflect FDA-approved products supported by peer-reviewed clinical evidence; in some cases, approval is based on accelerated or non-Phase 3 data as specified in the cited studies (for example, lifileucel under FDA, accelerated approval based on the C-144–01 single-arm Phase 2 cohort). Long-term melanoma overall survival data for ipilimumab refer to the published 10-year follow-up of the pooled ipilimumab analysis. Data-source categories are as defined in Section 1.1. Abbreviations: ADC, antibody-drug conjugate; B-ALL, B-cell acute lymphoblastic leukemia; DLBCL, diffuse large B-cell lymphoma; dMMR, mismatch-repair-deficient; HCC, hepatocellular carcinoma; HNSCC, head and neck squamous cell carcinoma; mCRPC, metastatic castration-resistant prostate cancer; MSI, microsatellite instability; NSCLC, non-small cell lung cancer; ORR, objective response rate; OS, overall survival; PFS, progression-free survival; RCC, renal cell carcinoma; TIL, tumor-infiltrating lymphocyte; TNBC, triple-negative breast cancer.

**TABLE 3:** Convergent technology strategies integrating diagnostics, prevention, and treatment.

Strategy	Components	Diagnostic integration	Rationale	Development stage
NIR-PIT plus ICI	Cetuximab-IR700 plus anti-PD-1	ctDNA for response monitoring	Immunogenic cell death may convert cold tumors to hot; ICI may sustain response [63]	Phase 1/2 single-arm clinical observation (preliminary data)
PDT plus CRISPR CAR-T	GQD-PDT plus PD-1 knockout CAR-T	Spatial transcriptomics for TME mapping	PDT generates ROS; CAR-T may eliminate residual disease	Conceptual and preclinical (not yet in clinical testing)
Triple modality	NIR-PIT plus CAR-T plus mRNA vaccine	Liquid biopsy plus AI radiomics for adaptive dosing	Immunogenic cell death plus engineered T cells plus adaptive immune training	Conceptual; no clinical data
CRISPR antiviral plus mRNA vaccine	Cas9 mRNA plus antigen mRNA in lipid nanoparticles	Viral load monitoring via ctDNA or ctRNA	Disrupt viral oncogenes plus prevent reinfection [26]	Preclinical
Quantum-optimized photosensitizer plus ICI plus CAR-T	AI- or computationally designed photosensitizer plus checkpoint-resistant T cells	Multi-omic profiling plus AI treatment selection	Precision photosensitizer plus multi-gene-edited T cells	Future concept; no experimental validation
Closed-loop adaptive therapy	ctDNA monitoring plus AI plus adaptive dosing	Longitudinal liquid biopsy as feedback loop	Dynamic therapy adjustment based on molecular response [76]	Early clinical pilots; not prospectively validated as a closed-loop system

“FDA, approved” indicates Premarket Approval or New Drug Application approval. “Conditionally approved (Japan)” refers to PMDA, conditional early approval pending confirmatory data. “Preliminary single-arm” indicates early-phase, non-randomized data not sufficient to support regulatory or therapeutic conclusions. Abbreviations: ICI, immune checkpoint inhibitor; NIR-PIT, near-infrared photoimmunotherapy; NP, nanoparticle; PDT, photodynamic therapy; PTT, photothermal therapy; QD, quantum dot; ROS, reactive oxygen species.

## 2 The hallmarks framework: historical context and limitations

### 2.1 Evolution of the framework

The framework of cancer hallmarks has undergone four significant revisions. The initial 2000 version delineated six acquired functionalities of cancer cells: autonomy in growth signals, resistance to antigrowth signals, evasion of apoptosis, limitless replicative capacity, sustained angiogenesis, and tissue invasion and metastasis [2]. The 2011 revision incorporated two emerging hallmarks (deregulation of cellular energetics and immune evasion) and two enabling features (genome instability and tumor-promoting inflammation) [3]. The 2022 update introduced the acquisition of phenotypic plasticity and disrupted cellular differentiation as hallmark capacities, along with non-mutational epigenetic reprogramming, polymorphic microbiomes, and the presence of senescent cells as supplementary dimensions [4]. The 2026 revision consolidates these aspects into an integrative model comprising four interconnected dimensions: nine hallmark capabilities, five enabling characteristics, seven classes of hallmark-facilitating cells within the tumor microenvironment, and systemic interactions with the body in which the cancer arises [5].

### 2.2 Why the framework alone is insufficient

The hallmarks framework has been instrumental in organizing tumor biology; however, recent advancements have demonstrated its limitations as a guide for clinical innovation. Firstly, many of the most promising diagnostic and therapeutic technologies are agnostic to hallmarks. Liquid biopsy detects cancer using circulating nucleic acids, regardless of the hallmarks involved. AI-assisted imaging classifies tumors based on pattern recognition rather than mechanism. Messenger RNA vaccines stimulate immune responses against neoantigens without requiring knowledge of the specific hallmark categories from which they originate. Secondly, cancer biology is increasingly comprehended through continuous, multi-omic data streams rather than through the discrete categories imposed by the hallmarks. Thirdly, the most significant clinical advancements, including tumor-agnostic regulatory approvals, pan-cancer screening, and adaptive therapy, fundamentally transcend the boundaries of hallmark features. Therefore, this review considers the hallmarks as a vital biological context, while emphasizing the enabling technologies that currently propel clinical innovation (Figure 2).

## 3 Next-generation cancer diagnostics: redefining detection and classification

### 3.1 Multi-omic liquid biopsy platforms

Liquid biopsy has emerged as a primary driver of cancer diagnostics, enabling noninvasive detection, real-time monitoring, and prediction of treatment response through analysis of circulating biomarkers. Modern multi-omic liquid biopsy platforms integrate several analyte classes. Circulating tumor DNA (ctDNA) analysis detects somatic mutations, copy number alterations, and microsatellite instability from cell-free DNA fragments shed by tumors into the bloodstream, with detection sensitivities now approaching 0.01 percent variant allele frequency through error-corrected next-generation sequencing and molecular barcoding [6, 7]. Circulating tumor RNA provides information on gene expression, alternative splicing, and fusion transcripts that ctDNA cannot capture. Exosome-based assays isolate tumor-derived extracellular vesicles that carry proteins, lipids, and nucleic acids reflective of the tumor molecular state. Tumor-derived DNA methylation signatures can be analyzed in cell-free DNA: the Galleri multi-cancer early detection test (GRAIL) analyzes methylation patterns to identify tissue of origin and detect over 50 cancer types from a single blood draw [8].

The Food and Drug Administration (FDA) has granted Premarket Approval to FoundationOne Liquid CDx (P190032) and Guardant360 CDx (P200010) as companion diagnostics for specific targeted therapies [9, 10]. Multi-cancer early detection tests are currently undergoing regulatory review. The Galleri test was granted FDA Breakthrough Device Designation in 2018 and has been evaluated in the NHS-Galleri trial involving approximately 140,000 participants in the United Kingdom (ClinicalTrials.gov NCT05611632), as well as in the PATHFINDER 2 study with approximately 25,490 participants in the United States. On 29 January 2026, GRAIL submitted a modular Premarket Approval (PMA) application to the FDA for Galleri [11]. As of the date of this report, neither Galleri nor any other multi-cancer early detection test has received FDA premarket approval, 510(k) clearance, or *de novo* authorization for population-level screening. Evidence for mortality reduction remains an active area of investigation within the NHS-Galleri trial and other prospective studies. Pan-cancer screening approaches, such as CancerSEEK, identify cancer by combining signals, including mutations, methylation patterns, and protein biomarkers, that reflect disease presence rather than specific hallmark mechanisms [12].

**FIGURE 3:**
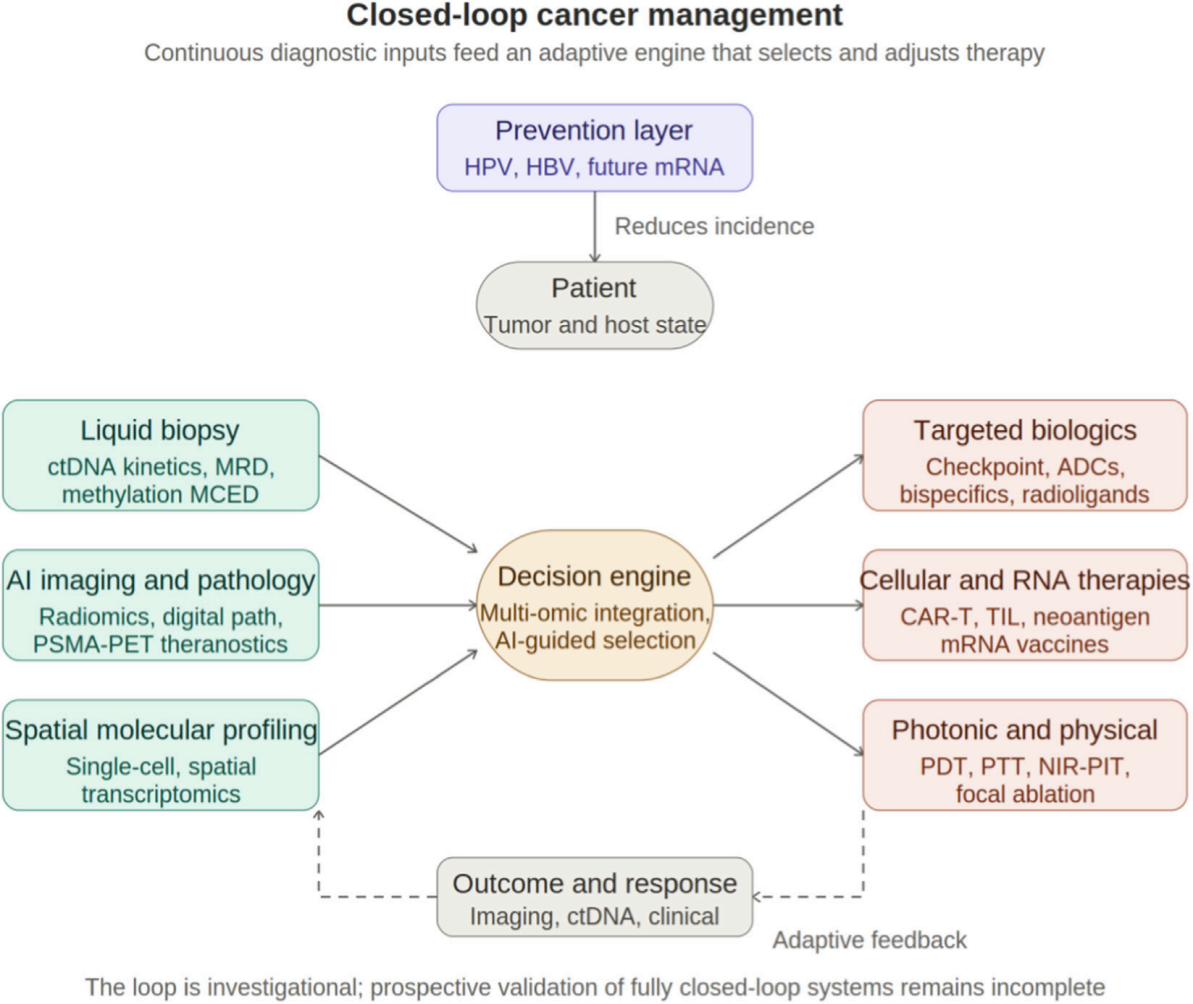
Illustrates the concept of closed-loop cancer management as an integrated framework. The diagram depicts the architecture of the closed-loop oncology system, which this review considers the ultimate destination of contemporary technological convergence. The patient is positioned at the center, with the prevention layer (including HPV, HBV, and forthcoming mRNA prevention) illustrated upstream as the primary intervention opportunity. Three diagnostic input streams are shown entering from the left: liquid biopsy involving continuous ctDNA kinetics, minimal residual disease (MRD) detection, and methylation-based multi-cancer early detection; artificial intelligence (AI)-based imaging and pathology, encompassing radiomics, digital pathology, and PSMA-PET theranostics; and spatial molecular profiling leveraging single-cell and spatial transcriptomic platforms. These data streams converge into a central decision engine that multi-omicsintegrates integration and selects AI-guided therapies. The engine directs treatment outputs across three modality categories displayed on the right: targeted biologics (such as checkpoint inhibitors, antibody-drug conjugates, bispecific engagers, and radioligands), cellular and RNA therapies (including CAR-T, TIL, and neoantigen mRNA vaccines), and photonic and physical therapies (such as photodynamic therapy (PDT), photothermal therapy (PTT), near-infrared photoimmunotherapy (NIR-PIT), and focal ablation). Outcomes and response data (encompassing imaging, ctDNA, and clinical information) are fed back through dashed adaptive-feedback arrows to update the diagnostic streams and re-train the decision engine. Color coding aligns with Figures 1, 2, using teal for diagnostics, purple for prevention, and coral for treatment. This figure is conceptual and not representative of any deployed product. As of the date of this review, prospective validation of fully closed-loop adaptive cancer management systems remains incomplete; therefore, the figure should be regarded as a research target rather than as an indication of current standard practice. Created in BioRender. Niazi, S. (2026) https://BioRender.com/q0lvwth.

A distinct and rapidly maturing diagnostic mode applies machine learning to population-scale plasma proteomics to estimate future cancer risk years before clinical presentation, rather than detecting tumor-derived material at the time of sampling. Using baseline Olink plasma proteomic profiles from the United Kingdom Biobank, Pandya et al. [13] trained a model that identified a 14-protein signature predicting incident lung cancer a median of more than 5 years before diagnosis, with replication across eight external cohorts and a held-out area under the curve of 0.865 when combined with patient characteristics, exceeding established clinical risk models such as the Liverpool Lung Project version 3 and the Lung Cancer Risk Assessment Tool. Unlike multi-cancer early detection assays that depend on tumor-derived signals and therefore on sufficient lesion burden, this signature reflected a tumor-promoting inflammatory state in the alveolar niche, was elevated in smokers and individuals exposed to particulate matter, was not associated with tumor stage, and did not decline after surgical resection, indicating that it captures host susceptibility and early tumor promotion rather than established malignancy. This risk-prediction strategy is hallmark-agnostic, in the same sense as liquid biopsy and AI imaging: it classifies individuals based on data-driven proteomic patterns linked to tumorigenic microenvironmental states, rather than any single hallmark capability. The approach generalizes beyond the lung. Papier et al. [14] reported 618 protein-cancer associations across 19 cancer types in the United Kingdom Biobank, of which 107 remained detectable in cases diagnosed more than 7 years after blood draw, and several were supported by concordant genetic evidence (cis-pQTL and exome-wide protein genetic scores), suggesting that organ-specific or pan-cancer plasma proteomic risk signatures could extend molecular risk stratification across the cancer spectrum. Such signatures occupy a complementary position to methylation-based multi-cancer early detection, addressing risk prediction and prevention-trial enrichment rather than the detection of prevalent disease (Tables 2-7) (Figure 3).

**TABLE 4:** Next-generation diagnostic technologies in cancer.

Technology	Key analytes	Clinical applications	Regulatory status (April 2026)	Representative platforms
ctDNA liquid biopsy	Somatic mutations, CNAs, MSI	Companion diagnostics, MRD monitoring, therapy selection	FDA approved (PMA): FoundationOne liquid CDx (P190032); Guardant360 CDx (P200010)	Foundation medicine, guardant health, tempus
Methylation-based MCED	cfDNA methylation signatures	Pan-cancer early detection, tissue-of-origin prediction	Breakthrough device designation (galleri, 2018); GRAIL completed modular PMA submission 29 Jan 2026; not yet approved	Galleri (GRAIL), CancerSEEK (research)
AI radiomics	Quantitative imaging features	Tumor grading, molecular subtype prediction, response prediction	Selected FDA-cleared products; most academic tools research use only	Tempus, PathAI, academic tools
Digital pathology with AI	Histomorphological patterns	Automated grading, biomarker prediction from H&E	FDA *de novo* authorized (paige prostate, 2021)	Paige, PathAI, ibex medical analytics
Spatial transcriptomics	Spatially resolved gene expression	TME mapping, immune infiltration, clonal architecture	Research use only; clinical translation in progress	10x Genomics visium, MERFISH, slide-seq
Single-cell multi-omics	Transcriptome, epigenome, proteome per cell	Therapy resistance, rare subclone identification, MRD	Research use only; clinical translation in progress	10x Genomics chromium, BD rhapsody, CITE-seq
Plasma proteomic risk-prediction signatures	Multiplex plasma protein panels (olink, SomaScan)	Future cancer risk prediction (years before diagnosis); prevention-trial enrichment and stratification	Research use only; lung signature validated across eight cohorts; cross-cancer associations reported	14-Protein lung signature [13]; 19-cancer panel [14]

Where data source is listed as company-reported, the cited results derive from manufacturer press releases or investor communications and have not yet been published in peer-reviewed journals. Such data should be interpreted as preliminary signals of feasibility rather than as evidence of established efficacy. Abbreviations: CR, complete remission; NHL, non-Hodgkin lymphoma; ORR, objective response rate; TCR, T cell receptor; TIL, tumor-infiltrating lymphocyte.

**TABLE 5:** Established and emerging immunotherapeutic modalities in cancer.

Modality	Representative agents	Approved indications	Key clinical evidence	Regulatory status
Anti-PD-1/PD-L1	Pembrolizumab, nivolumab, atezolizumab, durvalumab	NSCLC, melanoma, RCC, urothelial, HNSCC, MSI-high or dMMR (tissue-agnostic), more than 20 tumor types	KEYNOTE and CheckMate trials; durable OS benefit in multiple settings [30]	FDA approved (multiple agents and indications)
Anti-CTLA-4	Ipilimumab, tremelimumab	Melanoma, RCC, NSCLC, HCC, mesothelioma (in combinations)	Long-term melanoma OS data; combination with anti-PD-1 [36]	FDA approved (multiple combinations)
Anti-LAG-3	Relatlimab plus nivolumab	Melanoma (first-line)	RELATIVITY-047: PFS benefit over nivolumab alone [33]	FDA approved (March 2022)
ADC (HER2)	Trastuzumab deruxtecan	HER2-positive and HER2-low breast, gastric, NSCLC	DESTINY-Breast03 and DESTINY-Breast04 [37, 38]	FDA approved
ADC (Trop-2)	Sacituzumab govitecan	Metastatic TNBC, urothelial carcinoma	ASCENT trial [39]	FDA approved
Bispecific T-cell engager	Teclistamab, glofitamab, epcoritamab, blinatumomab	Multiple myeloma, DLBCL, B-ALL	MajesTEC-1 [42]; NP30179 [44]	FDA approved (multiple, 2022–2024)
Radioligand (PSMA)	Lu-177 PSMA-617 (pluvicto)	mCRPC (PSMA-positive)	VISION trial: OS benefit [46]	FDA approved (March 2022)
TIL therapy	Lifileucel (amtagvi)	Unresectable or metastatic melanoma	C-144-01: 31.5 percent ORR [49]	FDA accelerated approval (February 2024)

Data sources are graded as follows. Peer-reviewed: full primary publication in an indexed journal. Press release: company-reported data not yet peer-reviewed. Conference data: presented at a scientific meeting (oral or poster) but not yet published as a full paper. Trial registry: information from ClinicalTrials.gov or equivalent. The 5-year intismeran autogene data (49 percent risk reduction) supersede the earlier 3-year figures (44 percent recurrence reduction, 65 percent distant metastasis reduction) for current reporting purposes; both are included for historical completeness. Abbreviations: HNSCC, head and neck squamous cell carcinoma; ORR, objective response rate; PD-1, programmed cell death protein 1.

**TABLE 6:** Quantum-dot-based photosensitizers for cancer photonic therapy (preclinical research).

QD type	Reported singlet oxygen quantum yield	Mechanism	Notable properties	Development stage
Graphene QDs	Approximately 1.3 (*in vitro* [54])	Multistate sensitization (S1 plus T1)	Highest reported quantum yield in source; broad absorption; biocompatible in cell culture	Preclinical only
HPGQD (D-12 A)	0.85 (*in vitro* [55])	D-12 A donor-acceptor architecture	Rapid ROS generation; self-quenching reported at 10 min	Preclinical, *in vitro* and *in vivo*
Se-decorated GQDs plus methylene blue	Enhanced versus methylene blue alone [65]	FRET from GQDs to methylene blue; enhanced intersystem crossing	Afterglow potentially useful for deep tumors; combination approach	Preclinical
Carbon QDs	Variable; nitrogen-doped CQDs reported superior [64]	Type I and type II; up-conversion	Tunable photoluminescence; low cost; ease of functionalization	Preclinical
GQD-porphyrin exciplexes	95 percent energy transfer efficiency reported [66]	Resonance energy transfer	Enhanced porphyrin properties; cellular uptake	Preclinical

All entries in this table are preclinical. Reported quantum yields are typically from *in vitro* measurements and may not translate directly to *in vivo* therapeutic settings. None of the listed quantum-dot photosensitizers has entered clinical trials. Abbreviations: CQD, carbon quantum dot; FRET, forster resonance energy transfer; GQD, graphene quantum dot; QD, quantum dot; ROS, reactive oxygen species.

**TABLE 7:** Comparison of photonic cancer treatment modalitie.

Modality	Photophysical basis	Key agents	Clinical status (April 2026)	Effect on tumor biology
PDT (type I)	Electron transfer from triplet excited state to substrate; free radical generation	Porfimer sodium, temoporfin, BODIPY derivatives	FDA approved (multiple indications) [53; 57]	Cell death, vascular disruption, metabolic stress
PDT (type II)	Energy transfer from triplet excited state to ground-state molecular oxygen generating singlet oxygen	Verteporfin, graphene QDs (preclinical), carbon QDs (preclinical)	FDA approved (verteporfin, ophthalmologic; porfimer sodium, oncologic); QDs preclinical only [54; 55]	Cell death, inflammation, immunogenic cell death
PTT (NIR-i)	Nonradiative relaxation; phonon-mediated heating (700–1,000 nm)	Gold nanoshells (AuroShell), indocyanine green	Clinical trials (NCT02648035); not FDA approved	Cell death, vasculature
PTT (NIR-II)	Deep-tissue heating (1,000–1700 nm); reduced scattering	Carbon nitride NPs, organic polymers	Preclinical and early clinical [58]	Cell death, vasculature, invasion
NIR-PIT	Photoinduced ligand release; physical membrane disruption; immunogenic cell death	Cetuximab-IR700 (akalux)	Conditionally approved in Japan (2020); phase 3 ongoing globally [60]	Immune evasion reversal, inflammation, plasticity
Combined PDT and PTT	Synergistic ROS generation plus hyperthermia	Multifunctional nanoparticles	Preclinical only [58]	Multiple effects simultaneously
NIR-PIT plus ICI	Immunogenic cell death plus checkpoint blockade	Cetuximab-IR700 plus pembrolizumab	Phase 1/2 (n = 9 evaluable; preliminary single-arm) [63]	Immune evasion reversal plus multiple

Development stage descriptors are defined as follows. Conceptual: described in commentary or perspective articles without experimental data. Preclinical: experimental data in cell culture or animal models, no human data. Phase 1/2 single-arm: human safety and feasibility data only, no controlled comparison. Early clinical pilots: small prospective cohorts without prospective randomized validation. None of the strategies in this table is FDA-approved or has been validated in randomized Phase 3 trials. Abbreviations: CAR, chimeric antigen receptor; ctDNA, circulating tumor DNA; ctRNA, circulating tumor RNA; GQD, graphene quantum dot; ICI, immune checkpoint inhibitor; NIR-PIT, near-infrared photoimmunotherapy; PD-1, programmed cell death protein 1; PDT, photodynamic therapy; ROS, reactive oxygen species; TME, tumor microenvironment.

### 3.2 AI-assisted radiomics and digital pathology

Artificial intelligence is revolutionizing cancer imaging and pathology by shifting from qualitative, observer-dependent disciplines to quantitative, reproducible diagnostic platforms. Radiomics systematically extracts high-dimensional quantitative features from medical images, such as CT, MRI, and PET scans, including texture, shape, intensity distributions, and spatial relationships, which are often imperceptible through visual inspection [15]. Additionally, deep learning models trained on radiomic features can effectively predict tumor grade, molecular subtype, treatment response, and prognosis, often demonstrating performance that matches or surpasses that of expert radiologists across various cancer types [16].

Digital pathology, enabled by whole-slide imaging and convolutional neural networks, has resulted in the development of diagnostic applications authorized by the U.S. Food and Drug Administration (FDA). The Paige Prostate system received FDA *de novo* authorization as the inaugural AI-based pathology product approved for clinical implementation [17]. Foundation models trained on millions of histopathology images can recognize morphological patterns associated with specific genomic alterations, including predicting microsatellite instability status directly from hematoxylin and eosin-stained slides without the need for molecular testing [18]. These methodologies classify tumors based on data-driven patterns rather than mechanistic categories, thereby facilitating tumor-agnostic biomarker identification and pan-cancer diagnostics.

### 3.3 Spatial transcriptomics and single-cell sequencing

Spatial transcriptomics and single-cell RNA sequencing offer unparalleled resolution for understanding tumor heterogeneity and the architecture of the microenvironment. Techniques such as 10x Genomics Visium, MERFISH, and Slide-seq map gene expression profiles to exact spatial locations within tissue sections, elucidating how various cell populations interact *in situ* [19, 20]. Single-cell multi-omic methodologies that concurrently measure transcriptome, epigenome, and proteome at the single-cell level facilitate the identification of rare cell states, therapy-resistant subclones, and immune cell phenotypes within the tumor microenvironment.

These technologies are clinically actionable because they enable real-time monitoring of clonal evolution under therapeutic pressure, identification of minimal residual disease, and prediction of therapy resistance before clinical progression. Incorporation of these data streams into clinical decision-making is being facilitated by cloud-based bioinformatics platforms and standardization efforts coordinated through initiatives such as the Human Tumor Atlas Network [21].

### 3.4 Regulatory validation and clinical integration

The clinical adoption of next-generation diagnostics necessitates regulatory validation, reimbursement pathways, and integration into routine healthcare practices. The Food and Drug Administration (FDA) has implemented regulatory frameworks for companion diagnostics, laboratory-developed tests, and increasingly, artificial intelligence/machine learning (AI/ML)-based software as medical devices [22]. The European Union *In Vitro* Diagnostic Regulation has introduced more stringent requirements concerning clinical evidence. Key challenges encompass demonstrating clinical utility, beyond mere analytical validity, addressing false-positive rates in asymptomatic screening populations, establishing evidence-based follow-up algorithms for positive multi-cancer early detection results, and ensuring equitable access.

A note on regulatory terminology used throughout this review: “FDA approved” refers to full approval through Biologics License Application, New Drug Application, or Premarket Approval pathways; “FDA cleared” refers to 510(k) clearance for devices demonstrating substantial equivalence; “FDA *de novo* authorized” refers to *de novo* classification for novel low-to-moderate risk devices; “FDA accelerated approval” refers to approval under Subpart H or Subpart E based on surrogate endpoints with confirmatory trials required; “Breakthrough Device Designation” is a regulatory pathway designation, not an approval; “PMA submitted” indicates that a Premarket Approval application has been filed but not yet approved; “conditionally approved (Japan)” refers to approval under the Pharmaceuticals and Medical Devices Agency conditional early approval system requiring post-market confirmatory data; and “research use only” indicates technologies not yet cleared or approved for clinical diagnostic use.

## 4 Cancer prevention: from lifestyle modification to technology-enabled immunoprevention

### 4.1 Beyond lifestyle and environmental modifiers

Classical cancer prevention has traditionally concentrated on modifiable risk factors including tobacco cessation, dietary modifications, physical activity, sun protection, and the reduction of exposure to occupational and environmental carcinogens. Although these strategies retain their significance, the most impactful technological advancement to date is prophylactic vaccination against oncogenic viruses, which disrupts the process of oncogenesis at the molecular and immunological levels prior to the establishment of classical hallmarks.

### 4.2 Established prophylactic cancer vaccines

Prophylactic cancer vaccines constitute the most efficacious technological approach to prevention within the field of oncology. Human papillomavirus (HPV) vaccines, such as Gardasil 9 and Cervarix, effectively prevent infections caused by high-risk HPV strains responsible for nearly all cervical cancers, as well as a significant proportion of oropharyngeal, anal, vulvar, vaginal, and penile cancers. Data collected at the population level from Sweden and the United Kingdom illustrate substantial declines in the incidence of cervical intraepithelial neoplasia and invasive cervical cancer following the implementation of national vaccination initiatives, with vaccine-type HPV infections approaching eradication in vaccinated cohorts [23, 24]. The hepatitis B virus (HBV) vaccine serves to prevent chronic HBV infections, the primary etiological factor for hepatocellular carcinoma globally, and has demonstrated a reduction in liver cancer incidence in vaccinated populations over subsequent decades of observation [25].

These vaccines intervene in oncogenesis prior to the acquisition of any traditional hallmark. They inhibit the viral infection that would otherwise trigger persistent proliferative signaling (such as HPV E7 activation of CDK pathways), impede the inactivation of growth suppressors (such as HPV E6-mediated degradation of p53), and prevent genomic instability (such as HBV integration-induced chromosomal damage). The success of prophylactic cancer vaccines illustrates that preventive measures can be both mechanistically accurate and applicable on a population-wide scale.

### 4.3 Future directions in cancer prevention

In addition to the established use of viral-vector methods for cancer prevention, various innovative technological platforms are currently under investigation as potential future preventive tools. These methodologies are still in the research phase and have not yet undergone evaluation in prospective human cancer-prevention clinical trials. Consequently, they are presented herein as avenues for further research rather than as ready-to-implement preventive interventions.

Messenger RNA platforms in prevention. The mRNA vaccine platform, validated at scale during the COVID-19 pandemic, has demonstrated therapeutic activity against established cancers in early-phase studies (see Section 5.10) but has not yet entered clinical prevention trials in cancer. Theoretical extension to high-risk populations such as carriers of BRCA1/2, Lynch syndrome genes, or TP53 mutations would require demonstrating immunogenicity, long-term safety, and efficacy in healthy individuals at risk, prerequisites that have not yet been established. The current evidence base for mRNA cancer vaccines is therapeutic, not preventive.

**FIGURE 4:**
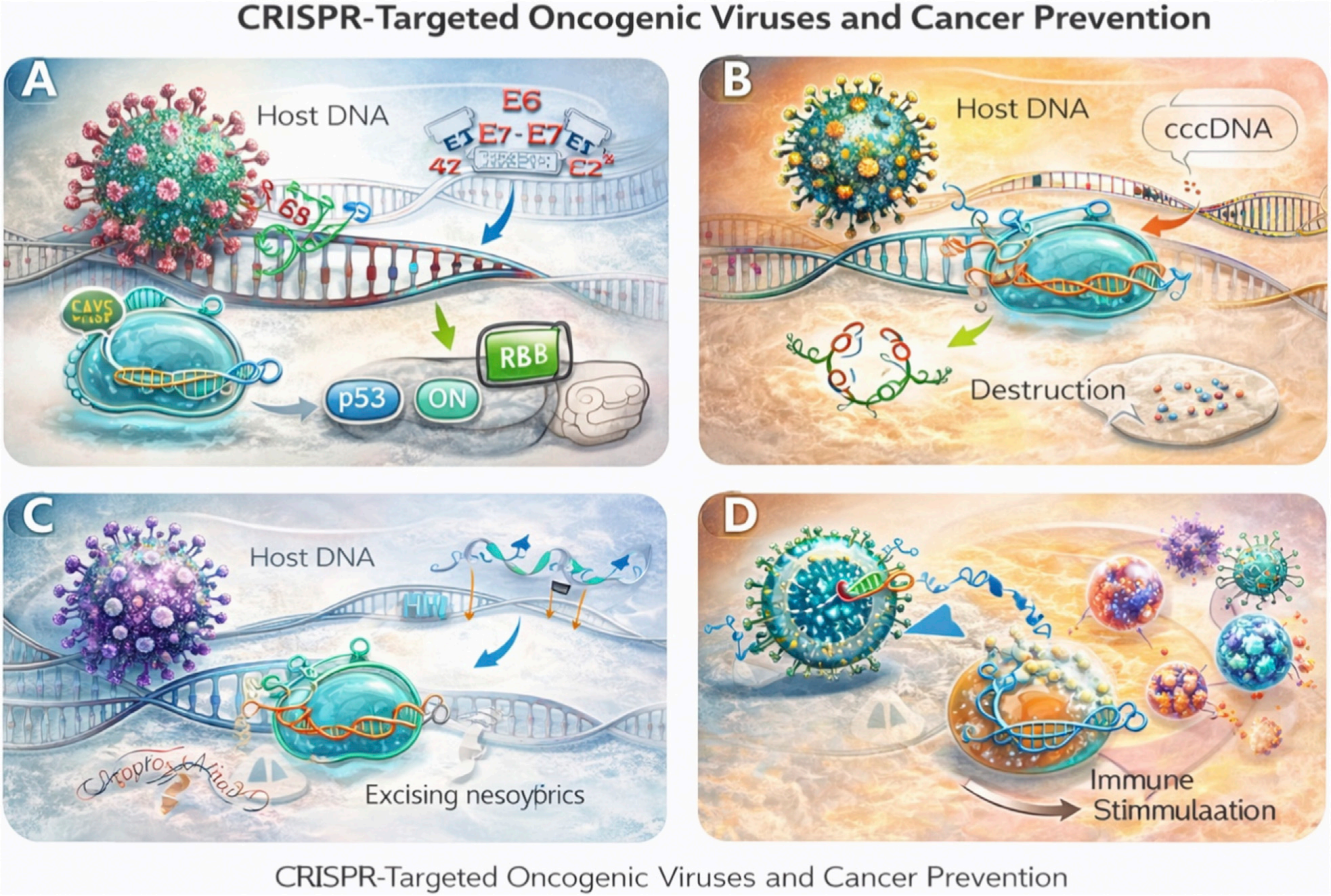
CRISPR-Targeted Oncogenic Viruses and Cancer Prevention. The four panels schematically illustrate complementary CRISPR-based strategies for preventing virus-driven cancers. Panel **(A)** Targeted disruption of HPV oncogenes (E6 and E7) using Cas9 to restore host p53 and RB tumor-suppressor function in infected cells. Panel **(B)** CRISPR-mediated destruction of HBV covalently closed circular DNA (cccDNA) in hepatocytes, depicted as cleavage and degradation of the viral episome. Panel **(C)** Precision excision of integrated viral oncogenic sequences from host DNA. Panel **(D)** mRNA-lipid nanoparticle delivery of CRISPR components combined with antigen-encoding mRNA, conceptually proposed as a single-vector preventive strategy that combines viral-genome editing with immune priming. All four approaches remain at preclinical or very early clinical stages of development; significant challenges related to delivery, off-target editing, and clinical safety must be addressed before any can be considered for human preventive application. The figure illustrates mechanisms rather than a description of any deployed product. Created in BioRender. Niazi, S. (2026) https://BioRender.com/q0lvwth.

CRISPR-Cas approaches against oncogenic viruses. CRISPR targeting of HPV E6 and E7 oncogenes has been demonstrated in preclinical models to restore p53 and RB tumor suppressor functions within HPV-positive premalignant lesions. Furthermore, CRISPR-mediated disruption of hepatitis B virus (HBV) covalently closed circular DNA (cccDNA) in hepatocytes has achieved over 80 percent suppression of viral antigen production in preclinical studies utilizing dual-guide-RNA techniques [26]. The delivery of CRISPR components via mRNA-lipid nanoparticle systems has been proposed on a conceptual basis for combined gene-editing and vaccine therapeutics. However, significant barriers to delivery, potential off-target effects, and the necessity for clinical safety validation must be addressed prior to the consideration of these approaches for human preventive applications [27]. Figure 4 schematically depicts the principal CRISPR-based strategies currently under preclinical investigation for the prevention of virus-induced cancers.

Shared neoantigen and personalized prophylaxis concepts. Shared neoantigen vaccines targeting common driver mutations such as KRAS G12D, TP53 R175H, and IDH1 R132H in genetically defined subgroups have been proposed as a middle ground between fully personalized and population-level prevention, supported by consortium efforts to improve the accuracy of neoantigen prediction [28]. Personalized neoantigen prophylaxis for individuals with hereditary cancer syndromes has been suggested on theoretical grounds. Neither approach has been validated in clinical prevention trials, and the immunological, safety, ethical, and efficacy prerequisites for translating these concepts into deployable strategies remain to be established.

Biomarker-stratified molecular interception. A complementary preventive paradigm is emerging that does not introduce a new antigen or editing agent but instead uses a circulating biomarker to identify the individuals and the temporal window in which an existing anti-inflammatory intervention can interrupt tumor promotion. The Canakinumab Anti-inflammatory Thrombosis Outcome Study (CANTOS) trial demonstrated a dose-dependent reduction in lung cancer incidence with interleukin (IL)-1β inhibition, yet the very high number needed to treat in an unselected population limited translation, and canakinumab showed no benefit in established non-small cell lung cancer. Pandya et al. [13] addressed this gap by showing, in a retrospective analysis of the CANTOS proteomic sub-cohort, that the 14-protein plasma signature identified the subgroup deriving preventive benefit: in participants with a high baseline signature, anti-IL-1β therapy reduced lung cancer incidence and lowered the number needed to treat from 1,516 in the low-signature group to 55 in the high-signature group, whereas no benefit was seen in low-signature individuals. Mechanistically, the same signature was inducible by particulate matter, oncogenic EGFR, and IL-1β, and IL-1β blockade restrained the expansion of keratin-8- and claudin-4-positive (KAC) alveolar transitional cells implicated in lung adenocarcinoma initiation, providing a biological rationale for a defined interception window that precedes detectable malignancy. This approach is preventive in intent yet diagnostic in mechanism, exemplifying the convergence of risk prediction and prevention discussed in Section 3.1. It remains exploratory: the signature-by-treatment interaction did not reach statistical significance, the analysis was retrospective in a trial enriched for cardiovascular rather than lung cancer risk, and prospective, signature-stratified prevention trials with serial sampling and absolute quantification are required before deployment. Extension to other cancers is plausible in principle, since organ-specific or pan-cancer plasma proteomic risk signatures have been described [14], but no analogous interception strategy has yet been validated outside the lung.

## 5 Treatment: modality-class organization beyond hallmark mapping

This section does not associate therapies with individual hallmarks. Instead, it categorizes treatment strategies by modality class. This approach reflects the evolving practice and regulation of oncology. Tumor-agnostic therapies (such as pembrolizumab for MSI-high tumors and larotrectinib for NTRK-fusion tumors), platform-based treatments, and adaptive regimens guided by real-time molecular feedback function independently of particular hallmark categories.

### 5.1 Immune checkpoint inhibitors: established backbone and evolving resistance strategies

Immune checkpoint inhibitors constitute the most significant advancement in immunotherapy over the past decade and now serve as the foundational treatment for numerous solid and hematologic malignancies. Monoclonal antibodies targeting programmed cell death protein 1 (pembrolizumab, nivolumab, cemiplimab), its ligand PD-L1 (atezolizumab, durvalumab, avelumab), and cytotoxic T-lymphocyte-associated protein 4 (ipilimumab, tremelimumab) have received approval from the U.S. Food and Drug Administration (FDA) across more than twenty tumor types and in multiple therapeutic lines, predominantly in the unresectable, locally advanced, or metastatic settings, with a growing number of approvals as adjuvant and neoadjuvant therapy in earlier-stage disease [29, 30]. The tissue-agnostic approvals of pembrolizumab for unresectable or metastatic MSI-high or mismatch-repair-deficient solid tumors (2017) and for unresectable or metastatic tumors with high tumor mutational burden (2020) exemplify a regulatory paradigm wherein biomarker status, rather than tissue of origin, determines treatment eligibility.

Despite significant therapeutic efficacy, primary and acquired resistance to checkpoint inhibitors remain prominent obstacles. Mechanisms include the loss of antigen presentation (B2M mutations, HLA downregulation), activation of alternative checkpoint pathways (LAG-3, TIGIT, TIM-3), immunosuppressive remodeling of the tumor microenvironment (myeloid-derived suppressor cells, regulatory T cells, TGF-beta signaling), and T cell exclusion mediated by the WNT/beta-catenin pathway [31, 32]. Next-generation checkpoint inhibitors targeting LAG-3 (relatlimab, FDA-approved in combination with nivolumab for unresectable or metastatic melanoma [33]), TIGIT, and TIM-3 are actively undergoing clinical evaluation. Combination strategies involving checkpoint inhibitors with chemotherapy, targeted therapy, anti-angiogenic agents, or novel immunomodulators have broadened the responsive patient population, as exemplified by the approvals of pembrolizumab combined with chemotherapy in metastatic non-small cell lung cancer [34], atezolizumab combined with bevacizumab in unresectable hepatocellular carcinoma [35], and nivolumab combined with ipilimumab in advanced (intermediate- or poor-risk) renal cell carcinoma [36]. The bulk of contemporary checkpoint-inhibitor evidence and indications, including the combinations cited above, has been generated in the metastatic, locally advanced, or unresectable disease setting, even though several agents have subsequently obtained adjuvant or neoadjuvant approvals in earlier-stage disease.

### 5.2 Antibody-drug conjugates: rapidly expanding indications

Antibody-drug conjugates have emerged as one of the fastest-growing therapeutic classes in oncology, combining the target specificity of monoclonal antibodies with the cytotoxic potency of small-molecule payloads. The field has been transformed by advances in linker chemistry, payload optimization, and target selection. Trastuzumab deruxtecan (Enhertu) targets HER2 and has been approved by the FDA for HER2-positive and HER2-low metastatic breast cancer based on the DESTINY-Breast03 and DESTINY-Breast04 trials, establishing HER2-low as a clinically relevant category [37, 38]. Trastuzumab deruxtecan has also been approved for locally advanced or metastatic HER2-positive gastric or gastroesophageal junction adenocarcinoma in patients who have received a prior trastuzumab-based regimen, and for previously treated metastatic HER2-mutant non-small cell lung cancer. Sacituzumab govitecan (Trodelvy), targeting Trop-2, is approved for metastatic triple-negative breast cancer and for locally advanced or metastatic urothelial carcinoma in patients with prior platinum-containing chemotherapy and a checkpoint inhibitor [39]. Enfortumab vedotin (Padcev), targeting Nectin-4, has demonstrated practice-changing efficacy in locally advanced or metastatic urothelial carcinoma in combination with pembrolizumab [40]. Additional antibody-drug conjugates in late-stage development target a growing range of antigens, including FolR-alpha, CEACAM5, Nectin-4, Claudin 18.2, and B7-H4, indicating continued rapid expansion of this therapeutic class.

### 5.3 Bispecific antibodies and T-Cell engagers

Bispecific antibodies that simultaneously engage tumor antigens and immune effector cells represent a rapidly advancing class of therapeutics. T-cell-engaging bispecific antibodies redirect cytotoxic T cells toward tumor cells by binding to CD3 on T cells and a tumor-associated antigen on malignant cells. Blinatumomab (targeting CD19 and CD3), the first bispecific T-cell engager approved by the FDA, has become an established treatment for relapsed or refractory B-cell acute lymphoblastic leukemia with minimal residual disease [41]. In hematologic malignancies, several bispecific antibodies targeting BCMA via CD3 have received FDA approval for relapsed or refractory multiple myeloma, including teclistamab [42], elranatamab, and talquetamab (targeting GPRC5D via CD3 [43]). Additionally, glofitamab (targeting CD20 via CD3) and epcoritamab (targeting CD20 via CD3) are approved for relapsed or refractory diffuse large B-cell lymphoma [44, 45].

In solid tumors, bispecific antibody development is advancing, with agents targeting DLL3, MUC16, PSMA, EGFR, and HER2 in various combinations with CD3 or other immune cell engagers. Bispecific checkpoint inhibitors targeting both PD-1 and CTLA-4, or PD-1 and VEGF, are in advanced clinical development. These modalities are mechanism-agnostic. They redirect immune effector function regardless of which hallmark capabilities the target tumor has acquired.

### 5.4 Radioligand therapies

Radioligand therapies deliver targeted radiation to tumor cells by conjugating a tumor-specific ligand to a radioactive isotope, thus enabling precise irradiation of disseminated disease while minimizing damage to normal tissues. Lutetium-177-PSMA-617 (Pluvicto) has received FDA approval for PSMA-positive metastatic castration-resistant prostate cancer based on the VISION trial [46] and is presently under evaluation in earlier treatment settings. Lutetium-177-DOTATATE (Lutathera) is authorized for advanced, progressive, somatostatin-receptor-positive gastroenteropancreatic neuroendocrine tumors, as established by the NETTER-1 trial [47]. Moreover, ongoing clinical trials are assessing radioligand therapies targeting additional tumor antigens, including fibroblast activation protein, HER2, and CXCR4, thereby broadening the applicability of this modality to a wider spectrum of tumor types.

### 5.5 Tumor-infiltrating lymphocyte therapy

The adoptive transfer of tumor-infiltrating lymphocytes signifies a significant advancement in cellular immunotherapy for solid tumors. Lifileucel (Amtagvi), a tumor-infiltrating lymphocyte therapy derived from a patient’s own tumor-resident T cells, received expedited approval from the U.S. Food and Drug Administration (FDA) in February 2024 for the treatment of unresectable or metastatic melanoma that has previously been managed with PD-1 blocking antibodies and, when BRAF V600 mutations are present, with a BRAF inhibitor, with or without an MEK inhibitor [48, 49]. In the pivotal C-144-01 trial (n = 73 in the efficacy-evaluable population), lifileucel exhibited an objective response rate of 31.5 percent, including complete responses, among heavily pretreated patients with a median of three prior lines of therapy. The durability of the response is particularly noteworthy, as many responders sustain their benefit beyond 18 months.

Tumor-infiltrating lymphocyte therapy is now under investigation in additional solid tumor types, including non-small cell lung cancer, head and neck squamous cell carcinoma, and cervical cancer. The manufacturing process requires surgical tumor resection, *ex vivo* lymphocyte expansion over approximately 22 days, and lymphodepleting conditioning chemotherapy prior to infusion. This approach validates the principle that endogenous, polyclonal antitumor immune responses can be harnessed for clinically meaningful benefit in solid tumors, complementing the engineered approaches described in subsequent sections.

### 5.6 CRISPR-edited cellular therapies and immune engineering

Immune engineering encompasses CRISPR-edited CAR-T and TCR therapies as well as engineered natural killer cells. CRISPR-Cas9 facilitates precise, multiplex genetic modifications that address the fundamental limitations of first-generation cellular immunotherapies, including T cell exhaustion, an immunosuppressive tumor microenvironment, manufacturing complexity, and limited efficacy in solid tumors [50, 51].

Allogeneic, or universal, CAR-T cells use CRISPR to enable off-the-shelf products derived from healthy donors. Disruption of endogenous T cell receptor genes (TRAC, TRBC) is intended to prevent graft-versus-host disease, and knockout of B2M or HLA genes is intended to reduce immune rejection. Multiple companies have advanced allogeneic CRISPR-edited products, including anti-CD19, anti-CD70, and PD-1 knockout variants. All clinical evaluations to date have been conducted in heavily pretreated, relapsed or refractory, advanced, or metastatic disease cohorts. Manufacturer-reported press-release data have described, for example, a complete remission lasting more than 3 years in a single patient with advanced renal cell carcinoma in a Phase 1 cohort of an anti-CD70 construct, and a 94 percent overall response rate in a Phase 1 trial of a PD-1 knockout CAR-T product in relapsed or refractory B-cell non-Hodgkin lymphoma (n = 16 evaluable). These data have not been published in peer-reviewed journals and should be interpreted as preliminary signals of feasibility rather than evidence of established efficacy. Validation in randomized, controlled trials is required.

**FIGURE 5:**
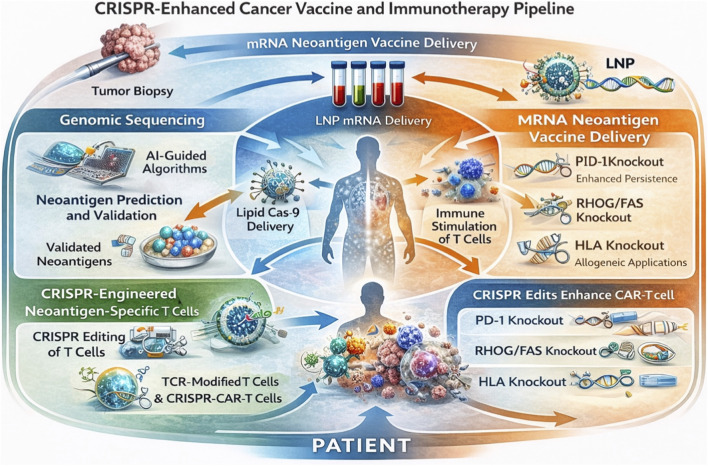
CRISPR-Enhanced Cancer Vaccine and Immunotherapy Pipeline. This schematic delineates the primary stages connecting tumor sampling to the deployment of engineered cellular and vaccine therapies. The process initiates with tumor biopsy and genomic sequencing of the patient’s tumor, followed by the application of AI-guided algorithms to predict candidate neoantigens. Subsequent CRISPR-based screening validates which predicted neoantigens evoke functional T-cell responses. Validated neoantigens are then allocated to two parallel pathways. In the vaccine pathway, these antigens are encoded into mRNA constructs and administered via lipid nanoparticles (LNP), with LNP-Cas9 delivery also illustrated for combined gene-editing and vaccination strategies. In the cellular therapy pathway, autologous T cells undergo CRISPR editing (including knockouts of PD-1, RHOG, FAS, and HLA to support persistence and resistance to exhaustion), and are engineered with tumor-specific receptors (TCR or CAR). Both pathways ultimately converge to stimulate immune responses and facilitate tumor cell eradication within the patient. The specified gene edits (e.g., PD-1 knockout for enhanced persistence; RHOG/FAS knockout for augmented antitumor activity; HLA knockout for allogeneic compatibility) are supported by peer-reviewed and preclinical evidence, as summarized in Section 5.6 and Table 1. This figure illustrates the process flow; however, not all steps are clinically validated, and several edits are currently in preclinical development. Created with BioRender. Niazi, S. (2026) https://BioRender.com/q0lvwth.

Checkpoint disruption in engineered T cells: CRISPR-mediated knockout of PD-1 (PDCD1), LAG-3, TIGIT, and other inhibitory receptors renders engineered T cells resistant to checkpoint-mediated exhaustion. Integrating the CAR construct into the PD-1 locus simultaneously introduces the receptor and disrupts the checkpoint, producing cells with enhanced cytotoxic activity in preclinical models. The CELLFIE platform, published in Nature in 2025, identified RHOG knockout as a potent CAR-T enhancer through genome-wide CRISPR screening, with combined RHOG and FAS double knockout enhancing antitumor activity across multiple *in vivo* models [52]. This finding is preclinical and has not yet entered clinical testing. Figure 5 schematically integrates these manufacturing and engineering steps into a unified CRISPR-enhanced cancer vaccine and cellular immunotherapy pipeline.

### 5.7 Photonic and photoimmunologic therapies

Photonic cancer treatments utilize interactions between light and matter to facilitate the targeted destruction of tumors with minimal collateral damage. Currently, three primary modalities are in clinical or late-preclinical development: photodynamic therapy, photothermal therapy, and near-infrared photoimmunotherapy. The mechanistic foundation of each modality can be elucidated through photophysics and photochemistry principles, without necessitating a separate quantum-mechanical explanation; the term “quantum yield” is employed here solely in its conventional photochemical context, defined as the ratio of beneficial photophysical events to the number of photons absorbed.

**FIGURE 6:**
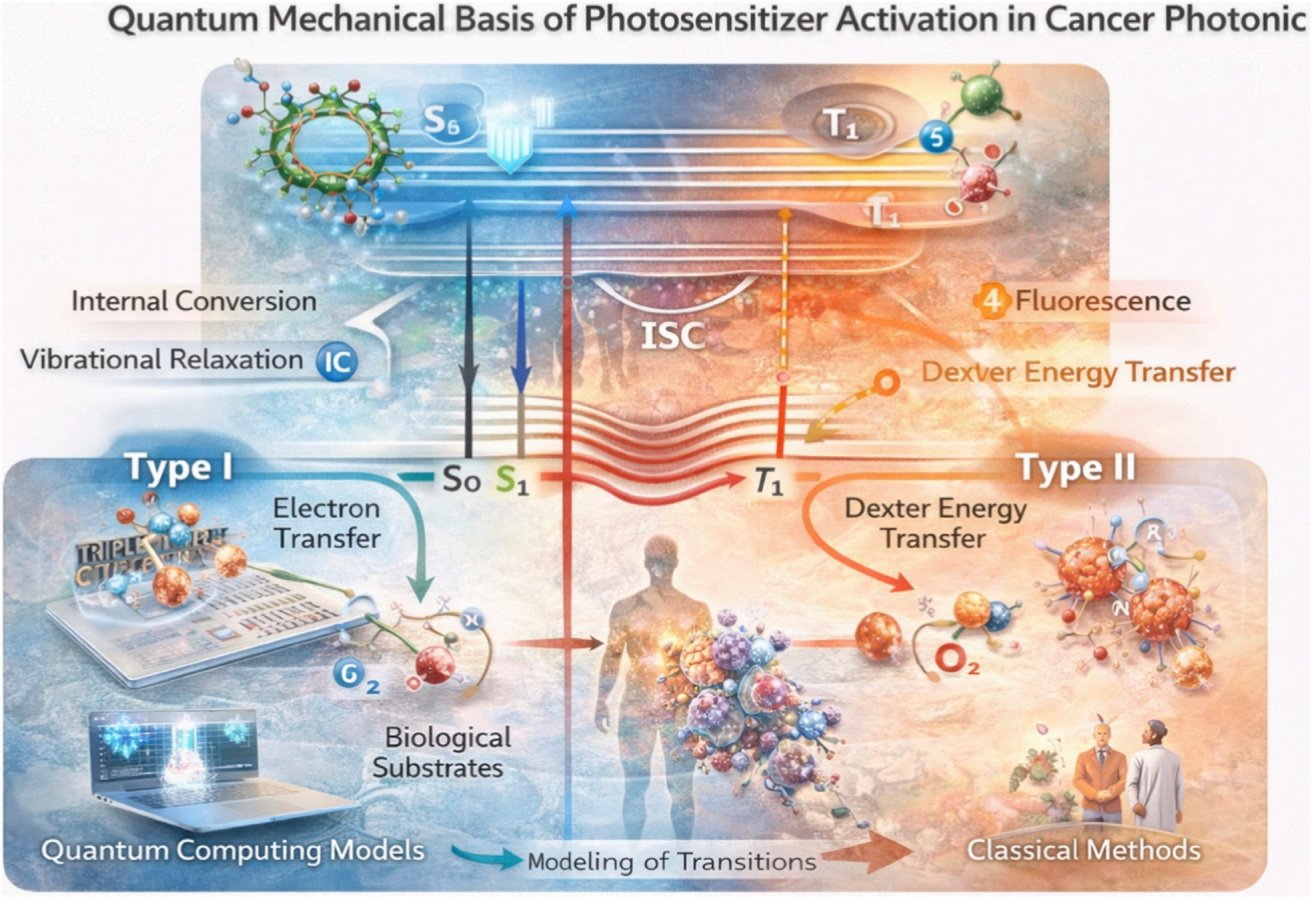
Photophysical Basis of Photosensitizer Activation in Photodynamic Therapy. The diagram constitutes a Jablonski-style schematic illustrating the electronic state transitions fundamental to photodynamic therapy. A photosensitizer absorbs a photon and transitions from the singlet ground state (S0) to a singlet excited state (S1). From S1, the molecule may undergo relaxation via fluorescence, internal conversion (IC) combined with vibrational relaxation, or through intersystem crossing (ISC) to a longer-lived triplet excited state (T1). From T1, two cytotoxic decay pathways become operational: a Type I pathway involving electron transfer to adjacent biological substrates, resulting in the formation of superoxide and hydroxyl radicals, and a Type II pathway involving Dexter energy transfer to ground-state molecular oxygen, leading to the generation of singlet oxygen. The lower panels illustrate that quantum chemical methodologies, encompassing both classical computational chemistry and emerging quantum computing approaches, can model these electronic transitions and thereby inform the design of photosensitizers. The term “quantum yield,” as used in the photosensitizer literature, denotes the ratio of beneficial photophysical events to the number of photons absorbed; the figure aims to depict photophysics and photochemistry rather than suggesting that quantum computing is currently a clinical tool. Applications of quantum computing in photosensitizer development are currently non-clinical and at conceptual or preliminary algorithmic stages. Created in BioRender. Niazi, S. (2026) https://BioRender.com/q0lvwth.

Photodynamic therapy uses photosensitizer molecules that absorb photons of an appropriate wavelength, undergo excitation to a singlet excited state, and transition through intersystem crossing to a longer-lived triplet excited state. Two cytotoxic pathways follow. The Type I pathway involves electron transfer to nearby substrates and generates superoxide and hydroxyl radicals. The Type II pathway involves energy transfer to molecular oxygen and generates singlet oxygen [53]. Figure 6 summarizes the photophysical basis of photosensitizer activation as a Jablonski-style diagram, showing the singlet ground state, singlet excited state, intersystem crossing to the triplet excited state, and the Type I and Type II decay pathways. Singlet oxygen quantum yield is the conventional efficacy parameter for photosensitizers, with values reported *in vitro* for graphene quantum dots and other nanostructured agents in the preclinical literature [54, 55]. Several photosensitizers have FDA approval for specific indications: porfimer sodium for esophageal cancer and endobronchial non-small cell lung cancer, temoporfin for advanced head and neck cancer, 5-aminolevulinic acid for actinic keratosis, and verteporfin for ophthalmologic use in age-related macular degeneration. Third-generation photosensitizers incorporating tumor-targeting moieties aim to achieve tumor-selective accumulation and activation [56, 57].

Photothermal therapy involves converting absorbed photon energy into heat through nonradiative relaxation processes, thereby elevating local tissue temperatures to induce either mild hyperthermia (42–48° C) or thermal ablation (above 50° C). Agents operating within the second near-infrared window (NIR-II, 1,000–1700 nm) offer enhanced tissue penetration and diminished scattering. Gold nanoshell-based photothermal ablation (AuroShell) has undergone evaluation in clinical trials for focal prostate ablation (NCT02648035). Additionally, carbon nitride nanoparticles have exhibited dual photothermal and photodynamic functionalities under a solitary 1,064 nm laser irradiation in preclinical models [58].

Near-infrared photoimmunotherapy employs antibody-photoabsorber conjugates such as cetuximab-IR700 (Akalux). Upon exposure to NIR light, these conjugates experience photoinduced ligand release, physically disrupting the cellular membrane and inducing rapid immunogenic cell death. The consequent release of damage-associated molecular patterns stimulates dendritic cells and has been documented to activate systemic antitumor immune responses in preliminary studies [59–61]. Akalux obtained conditional approval in Japan in September 2020 for the treatment of unresectable, locally advanced, or recurrent head and neck squamous cell carcinoma under the Japanese conditional early approval system; Phase 3 confirmatory trials are currently underway in the United States and internationally [62]. A modest Phase 1/2 study combining cetuximab-IR700 with pembrolizumab reported a high observed response rate in nine evaluable patients with recurrent head and neck squamous cell carcinoma. This is a single-arm, early-phase investigation that requires validation in larger, controlled clinical trials before definitive therapeutic conclusions can be drawn [63].

Quantum-dot photosensitizers, including graphene quantum dots, carbon quantum dots, and selenium-decorated graphene quantum dots, are currently under investigation in preclinical studies for multimodal therapeutic applications that combine photodynamic therapy, photothermal therapy, and imaging [64–66]. To date, no quantum-dot-based photosensitizer has advanced to clinical trials.

### 5.8 Computational and quantum approaches in drug and photosensitizer design

Computational chemistry, including density functional theory and molecular dynamics, remains the primary approach for developing photosensitizers and small-molecule therapeutics for oncology. Quantum computing has been proposed as a potential long-term supplement, capable of simulating strongly correlated electronic structure problems beyond the reach of classical computational methods. Currently, there are no clinical applications of quantum computing in the development of cancer therapeutics or photosensitizers. Recent resource estimates, based on fault-tolerant algorithm proposals, indicate requirements of approximately 180–350 logical qubits and Toffoli gate depths ranging from 10^7 to 10^9 for clinically relevant photosensitizer simulations [72]. Zehr et al. [73] review the current state of the field and conclude that quantum computing may eventually provide advantages for modeling the excited-state dynamics of photosensitizers. Present quantum hardware is substantially removed from the scale and fidelity necessary for clinically relevant simulations; the referenced resource estimates assume fault-tolerant quantum machines that are not yet available. Therefore, achieving practical quantum advantage in the design of photosensitizers or drugs remains several years to a decade away. Consequently, the development of cancer drugs and photosensitizers is anticipated to continue relying on classical computational chemistry and experimental validation in the foreseeable future.

### 5.9 RNA therapeutics beyond vaccines

The mRNA platform extends beyond vaccines to encompass a broader range of oncology RNA therapeutics. Messenger RNA-encoded cytokines, immune modulators, and tumor suppressors can be delivered via lipid nanoparticles to directly reprogram the tumor microenvironment. Small interfering RNA and antisense oligonucleotides can silence oncogenes or restore tumor suppressor gene expression with a level of specificity unattainable by small-molecule drugs. Patisiran (Onpattro) and givosiran (Givlaari) exemplify clinical precedents for lipid-nanoparticle-delivered siRNA therapeutics, although these are approved for indications outside oncology. Self-amplifying RNA constructs offer sustained antigen expression at lower doses, potentially reducing manufacturing demands. Circular RNA provides enhanced stability and prolonged protein expression. These RNA modalities constitute a platform-based therapeutic strategy that is inherently agnostic to hallmark classification: the same delivery technology can target proliferative signaling, immune evasion, metabolic reprogramming, or any other hallmark capability, depending solely on the encoded sequence. The majority of RNA therapeutic approaches for cancer remain in preclinical or early clinical development [74].

### 5.10 Therapeutic mRNA neoantigen vaccines

Therapeutic mRNA neoantigen vaccines represent the most clinically advanced application of the mRNA platform in oncology. The Phase 2 b KEYNOTE-942 trial assessed intismeran autogene (mRNA-4157, V940) in combination with pembrolizumab against pembrolizumab monotherapy in patients who have undergone complete resection of high-risk stage III/IV melanoma [68]. An earlier 3-year analysis, presented in a conference abstract, demonstrated a 44% reduction in recurrence and a 65% decrease in distant metastasis [67]. A pre-specified 5-year analysis, announced by Moderna and Merck in January 2026, indicated a sustained 49 percent reduction in the risk of recurrence or death (HR 0.510, 95 percent CI 0.294 to 0.887, one-sided nominal p = 0.0075; company disclosure, peer-reviewed analysis pending), compared to pembrolizumab alone [75]. Currently, the 5-year data are available through a corporate communication; a peer-reviewed publication is anticipated. Additionally, a Phase 3 confirmatory trial (V940-001, INTerpath program) is actively enrolling participants across melanoma and other tumor types.

Autogene cevumeran (BNT122) encodes up to 20 personalized neoantigens delivered as an RNA-lipoplex formulation. In a peer-reviewed Phase 1 study involving 16 patients with resected pancreatic ductal adenocarcinoma, the vaccine, in combination with atezolizumab and modified FOLFIRINOX, elicited *de novo* neoantigen-specific CD8^+^ T cell responses in 8 patients [69]. An updated follow-up at 3.2 years, published in Nature, confirmed the sustained persistence of neoantigen-specific T cells and a prolonged recurrence-free survival among immune responders (median not reached) compared with non-responders (median 13.4 months, p = 0.007) [70]. A randomized Phase 2 clinical trial in patients with resected pancreatic ductal adenocarcinoma is presently ongoing (NCT05968326). Other platforms demonstrating clinical activity include BNT111 in melanoma, TG4050 in head and neck cancer, and CVGBM in glioblastoma, each with varying levels of published evidence as summarized in Table 2.

### 5.11 Adaptive and platform-based therapies

AI-guided treatment selection involves the utilization of machine learning models that incorporate multi-omic tumor profiles, clinical data, and real-world outcomes to enhance precision in treatment decisions. Platforms such as Tempus, Foundation Medicine, and emerging AI-native oncology enterprises use deep learning techniques to align patients with suitable clinical trials and to forecast treatment responses. However, prospective validation of AI-guided treatment selection within randomized clinical trials remains limited, and the regulatory frameworks governing AI/ML-based clinical decision support are still undergoing development [22]. Addressing issues related to data bias, algorithmic transparency, and equitable access is essential before these tools can be adopted as a standard of care.

Closed-loop treatment systems: The integration of liquid biopsy monitoring, AI-assisted interpretation, and adaptive dosing algorithms offers the potential to modify therapy based on longitudinal biomarker data. The kinetics of circulating tumor DNA during treatment can inform decisions such as dose escalation, switching combination therapies, or discontinuing treatment. Preliminary clinical trials in colorectal cancer employing ctDNA-guided adjuvant therapy [76] and in non-small cell lung cancer support this concept; however, randomized validation is still underway. Notable validation gaps persist concerning the clinical utility of ctDNA-guided adaptive therapy across various tumor types, and challenges related to implementation, including turnaround time, assay standardization, and reimbursement, must be addressed. Figure 3 depicts the conceptual architecture of such a closed-loop cancer management system, illustrating how continuous diagnostic inputs inform an adaptive decision-making engine that selects and fine-tunes among the modality classes described in Sections 5.1 through 5.10. It is important to note that the figure is conceptual rather than representative of any currently deployed product; prospective validation of fully closed-loop systems remains incomplete.

Combination regimens guided by molecular feedback: Rational combination strategies are increasingly guided by real-time molecular data rather than solely by hallmark-based rationale. Approved combinations such as VEGF inhibitors in conjunction with immune checkpoint inhibitors for renal cell carcinoma [36], non-small cell lung cancer [77], and hepatocellular carcinoma [35]; venetoclax combined with hypomethylating agents for acute myeloid leukemia [78]; and enfortumab vedotin combined with pembrolizumab for urothelial carcinoma [40] exemplify how modality convergence fosters therapeutic innovation.

## 6 Discussion and conclusion

The hallmarks of cancer framework, currently in its fourth major iteration [5], has established a lasting vocabulary and rational structure for comprehending cancer biology. The preceding sections have summarized how progress in diagnostics, prevention, and treatment primarily results from convergent enabling technologies.

In diagnostics, multi-omic liquid biopsy platforms, AI-assisted radiomics, digital pathology, spatial transcriptomics, and single-cell sequencing are fundamentally transforming the methods for detecting, classifying, and monitoring cancer. Regulatory milestones, including the PMA-approved companion diagnostics (FoundationOne Liquid CDx, Guardant360 CDx), the Breakthrough Device Designation for multi-cancer early detection tests, the impending January 2026 modular PMA submission for Galleri, and *de novo* authorization for AI-based pathology (Paige Prostate), serve as evidence that technological advancements are spearheading a reorganization of cancer diagnostics. Ongoing investigations into clinical-utility validation, particularly regarding mortality reduction through multi-cancer early detection screening, remain a vital area of active research. In parallel, machine learning applied to population-scale plasma proteomics now predicts incident cancer years before clinical presentation, with a 14-protein signature forecasting lung cancer more than 5 years ahead and validated across eight cohorts [13], and with hundreds of protein-cancer associations spanning 19 cancer types reported across the United Kingdom Biobank [14]; this risk-prediction mode is distinct from tumor-derived detection and blurs the boundary between diagnostics and prevention.

Regarding preventive strategies, prophylactic vaccines for HPV and HBV have demonstrated efficacy in preventing oncogenesis at the population level [23–25]. The extension of mRNA vaccine platforms, CRISPR-enabled antigen optimization, and personalized neoantigen-based prophylaxis to high-risk populations is still in the investigational stage. None of these methods has been evaluated in human cancer prevention trials, and their discussion in this review is intended to highlight potential future directions. A near-term, mechanistically distinct preventive paradigm uses a circulating biomarker to stratify who benefits from an existing intervention: a plasma proteomic signature identified CANTOS participants in whom anti-IL-1β therapy reduced lung cancer incidence, lowering the number needed to treat from 1,516 to 55 [13]. Although exploratory and not yet extended beyond the lung, this signature-stratified interception illustrates how risk-prediction biomarkers may enrich and rationalize future molecular cancer-prevention trials.

In treatment, the current therapeutic landscape is anchored by immune checkpoint inhibitors, which now have FDA approval across more than 20 tumor types (solid and hematologic), including tissue-agnostic indications [29]. Antibody-drug conjugates, bispecific T-cell engagers, and radioligand therapies have rapidly expanded in recent years, providing new modalities with distinct mechanisms of action and growing indication portfolios. The accelerated approval of lifileucel (Amtagvi) for melanoma validates adoptive tumor-infiltrating lymphocyte therapy in solid tumors [48]. The therapeutic mRNA neoantigen platform now has 5-year Phase 2 b evidence in resected melanoma (intismeran autogene, 49 percent reduction in recurrence or death), with Phase 3 confirmation pending. Peer-reviewed 3.2-year follow-up of autogene cevumeran in resected pancreatic ductal adenocarcinoma demonstrates that vaccine-induced T cell responses can be durable. CRISPR-edited cellular therapies show early-phase promise in both allogeneic CAR-T manufacturing and enhanced T cell function, although Phase 3 validation is required. Photonic and photoimmunologic therapies, RNA therapeutics, and computational drug-design approaches represent additional modalities at varying stages of clinical maturity.

Several challenges persist. The cost and manufacturing complexity associated with personalized vaccines have been documented in manufacturing literature to approximate approximately 100,000 USD per patient at the current scale, with reported ranges varying by platform and manufacturing site; however, costs are anticipated to decrease through process optimization. Quantum computing hardware capable of modeling complex photosensitizer excited-state dynamics at a clinically relevant scale has not yet been developed. The safety profiles of gene-editing therapies necessitate ongoing optimization to mitigate off-target effects and delivery challenges. Regulatory frameworks governing AI-guided treatment adaptation, closed-loop systems, and multi-cancer early detection are still under development. Manufacturing timelines for personalized mRNA vaccines have been reported to decrease from approximately 9 weeks–4 weeks in published analyses, although further reductions are necessary for broader clinical adoption. Additionally, data bias in AI models, equitable access to advanced diagnostics and therapies, and the need for prospective validation across diverse patient populations constitute further barriers to clinical implementation.

Collectively, these developments indicate that convergent technologies integrating diagnostics, prevention, and treatment into continuous, data-driven systems now constitute the guiding principle of modern oncology, complementing rather than supplanting the biological foundation established by the hallmarks. Achieving this potential will require prospective validation of emerging modalities, equitable-access frameworks, and regulatory adaptation at a pace commensurate with the underlying scientific advancements.
